# Cone beam computed tomography changes upon oral appliance therapy for adult patients with obstructive sleep apnea: A non-randomized clinical trial

**DOI:** 10.1097/MD.0000000000039923

**Published:** 2024-10-04

**Authors:** Lina Khasawneh, Noor Al Mortadi, Eslam Abu-Ishqair, Basheer Khassawneh, Karem H. Alzoubi

**Affiliations:** a Department of Prosthodontics, Faculty of Dentistry, Jordan University of Science and Technology, Irbid, Jordan; b Department of Applied Dental Sciences, Faculty of Applied Medical Sciences, Jordan University of Science and Technology, Irbid, Jordan; c Department of Radiology, Jordan University of Science and Technology, Irbid, Jordan; d Department of Internal Medicine, Jordan University of Science and Technology, Irbid, Jordan; e Department of Pharmacy Practice and Pharmacotherapeutics, University of Sharjah, Sharjah, United Arab Emirates; f Department of Clinical Pharmacy, Faculty of Pharmacy, Jordan University of Science and Technology, Irbid, Jordan.

**Keywords:** biomimetic oral device, mandibular advancement device, obstructive sleep apnea, oral appliance therapy

## Abstract

**Background:**

Obstructive sleep apnea (OSA) is caused by narrowing or obstruction of the airway lumen at single or multiple levels of the airway, starting from the nasal cavity up to the larynx. Oral appliance therapy for the management of OSA is prescribed as an alternative treatment option for patients with mild to moderate OSA who fail to adhere to Continuous Positive Airway Pressure (CPAP) therapy. Treatment with oral appliances addresses the craniofacial deficiencies that cause OSA by providing means to mandibular advancement and palatal expansion, thus opening the airways and potentially preventing airway collapse during sleep. Imaging the upper airway is employed to investigate the narrowing or the obstruction in the airway. Three-dimensional imaging modalities such as cone beam computed tomography (CBCT) allow for detecting obstructions before commencing treatment and for evaluating changes in the upper airway dimensions after treatment. To evaluate the effect of the biomimetic oral appliance therapy (BOAT) device on the airway measurements taken from a CBCT before and after treatment in correlation with the changes in the AHI.

**Trial design:**

A non-randomized clinical trial.

**Methods:**

About 17 patients with mild-moderate OSA (9 males, 8 females; age, mean [SD]: 45.76 [10.31]) underwent BOAT therapy. Subjects had 2 months of follow-up visits, including examinations for progress and adjustment of the appliances. The mean apnea–hypopnea index (AHI) with no appliance in the mouth before BOAT and after treatment was recorded. The midpalate screw mechanism of the appliance was advanced once per week. The subjects were asked to wear the appliance for 10 to 12 h/d and night. Pre and Post CBCT were taken. Paired *T*-test was used to analyze the results.

**Results:**

The treatment duration was 15.4 ± 6.3 months. Before treatment, at the diagnosis stage, the mean AHI of the sample (n = 17) was 24.0. After treatment, the mean AHI fell by 5% to 22.8% (*P* = .019), indicating enhanced upper airway functions. Airway measurements from the CBCT were not statistically significant despite improvement in the polysomnographic parameters.

**Conclusion:**

CBCT is a valuable tool for airway assessment and the determination of upper airway anatomic risk factors for OSA.

## 1. Introduction

The Wisconsin Sleep Cohort Study 1993 was a landmark study investigating obstructive sleep apnea (OSA). The reported prevalence of OSA in 1993 was 9% for females and 24% for males.^[1]^ However, due to the increase in the prevalence of obesity, the prevalence of OSA has increased over the last 2 decades; in 2013, the overall reported prevalence of OSA was 26% (34% among men and 17% among women).^[2]^ Patients presented with complaints of sleepiness and tiredness reported loud snoring and witnessed apnea.^[1]^ OSA leads to serious systemic cardiovascular consequences, namely, systemic hypertension.^[3]^ In addition, people with OSA are 3 times more likely to experience road traffic accidents.^[4]^

Clinical evaluation of airway obstruction relies on a multidisciplinary approach. Clinically, the Mallampati score, modified by Friedman, relies on observation of the position of the soft palate by asking the patient to open wide without protruding the tongue.^[5]^ Clinical observations, in addition to signs and symptoms, and a polysomnography (PSG) sleep study confirmed the diagnosis of OSA.

Obstructive sleep apnea is diagnosed by a PSG overnight sleep study prescribed by a licensed sleep physician. This test is considered the gold standard for diagnosing OSA.^[6]^ An apnea–hypopnea index (AHI) of 5 apneas per hour is a diagnostic indicator of OSA. Based on the AHI score, there are 3 levels for OSA: mild AHI ≤ 5, moderate AHI 5 to 15, and severe AHI ≥ 30.^[7]^

Obstructive sleep apnea is managed by continuous positive nasal airway pressure, which is considered the gold standard for treatment.^[8]^ It effectively prevents the collapse of the pharyngeal wall and maintains the airway open during sleep by maintaining a positive pharyngeal airway pressure so that it exceeds the surrounding pressure, thus maintaining the pharyngeal lumen open.^[8]^ However, despite continuous positive airway pressure (CPAP)’s efficacy in improving symptoms, adherence to CPAP treatment is suboptimal; 5% to 50% of patients who were prescribed CPAP either refused treatment or discontinued in the first week, and 12% to 25% of remaining patients discontinued CPAP treatment within 3 years.^[9]^

Oral appliance therapy is an alternative treatment to CPAP in the management of snoring in mild to moderate OSA patients who fail to adhere to or refuse CPAP treatment.^[10]^ It is favored as a simple, noninvasive treatment option. The oral appliance’s mechanism of action depends on mandibular protrusion or advancement in addition to repositioning the tongue to reduce airway collapse and widening the lateral aspects of the pharyngeal walls to improve airway patency and prevent airway collapse.^[11]^ The effective degree of advancement ranges from 6 to 10 mm or 65% to 70% of the maximum protrusion.^[12]^

Airway imaging modalities include magnetic resonance imaging (MRI), multidetector computed tomography (MDCT), and cone beam computed tomography (CBCT).^[13]^ Among these, CBCT is the most accessible to dentists. It has gained popularity in dentistry because it overcomes the drawbacks of 2-dimensional imaging, such as superimposition and the need to take 2 X-rays at different angles to detect pathology. In addition to the low radiation dose compared with MDCT, it was found that CBCT is a reliable tool for assessing the airway surrounded by soft tissue compared to MDCT.^[13]^ CBCT provides large-volume scans, typically including the cranial base, nasal cavity, nasopharynx, and oropharynx.^[14]^ The CBCT machine is small and affordable, and the acquisition time is considerably less than required for an MRI or an MDCT image.^[14]^ CBCT has several applications in dental sleep medicine. It is used to evaluate the airway in patients with OSA compared to healthy controls, assess treatment outcomes, and associate airway measurements with skeletal growth patterns.^[13]^ The purpose of this study is to evaluate the effect of the biomimetic oral appliance therapy (BOAT) device on the airway measurements taken from a CBCT before and after treatment in correlation with the changes in the AHI.

## 2. Methods

### 2.1. Patient selection and sampling method

**Figure 1.**
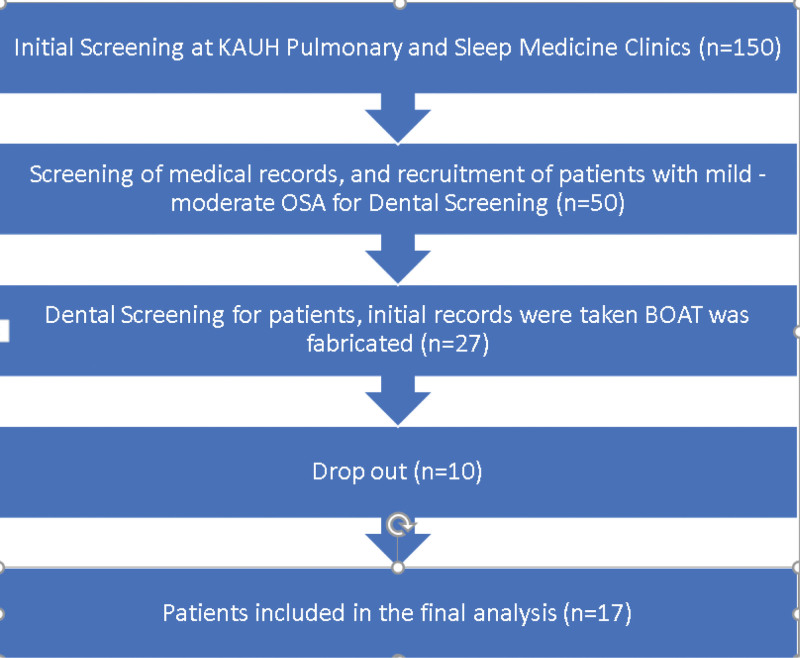
The study’s flow chart.

This is a secondary study of a previous work entitled “Arch Measurement Changes upon Biomimetic Oral Appliance Therapy for Adults with Obstructive Sleep Apnea.”^[15]^ Patients were recruited from King Abdullah University Hospital (KAUH) pulmonary and sleep medicine clinics. A total of 150 medical records of potentially suitable patients were screened. Fifty were invited for dental screening to determine their suitability for the BOAT treatment. Twenty-seven patients were suitable for treatment. Only 17 patients adhered to BOAT and were included in the analysis (Fig. 1). Inclusion criteria were patients who had a confirmed diagnosis of OSA by PSG. Patients who either refused CPAP treatment or failed to adhere to CPAP treatment were included in the study. Patients who had full dentition, good oral hygiene, good manual dexterity to use the expansion screw that comes with the BOAT device for expansion, and patients who were willing to attend follow-up appointments every 2 months. Exclusion criteria were patients who were unwilling to adhere to follow-up appointments, did not have good oral hygiene, and did not have sufficient teeth to retain the BOAT appliance.

### 2.2. Ethical approval

The study protocol was reviewed and approved by the institutional review board at Jordan University of Science and Technology (JUST) (IRB number is 15/108/2017, https://ClinicalTrials.gov identifier: https://clinicaltrials.gov/ct2/ show/NCT05087316). Study subjects were protected by following the Declaration of Helsinki and its amendments. The study protocol and procedures were explained to all participants, and all participants signed an informed consent form before participating in the study.

The study’s sample size was calculated using G-Power 3.1 (Universitat Kiel, Germany) based on the convenience sample method, large effect size, alpha value of 0.05, and power of 0.80. The minimum required number of subjects was 17.

### 2.3. Study protocol

Patients had a preoperative PSG at KAUH. The PSG was prescribed by a sleep physician (BK).

Patients were referred to the dental clinic in JUST for treatment with DNA device. A trained dentist (LK) who received training in BOAT carried out a thorough dental and craniofacial assessment before treatment. Scaling and polishing and oral hygiene instructions were given to patients. All necessary dental work was completed before commencing treatment. Preoperative CBCT was taken before treatment. Following the insertion of the DNA device, patients were recalled every 2 months for follow-up. After the study, postoperative CBCT and PSG were made to compare the results.

### 2.4. BOAT protocol

Alginate impressions for the fabrication of the customized DNA device. Maxillary and mandibular alginate impressions were taken. A protrusive bite record with an estimated 80% maximum protrusion was taken. All appliances were constructed at JUST dental clinics by a qualified dental technician specializing in removable orthodontic appliances who completed training in producing BOAT devices.

**Figure 2.**
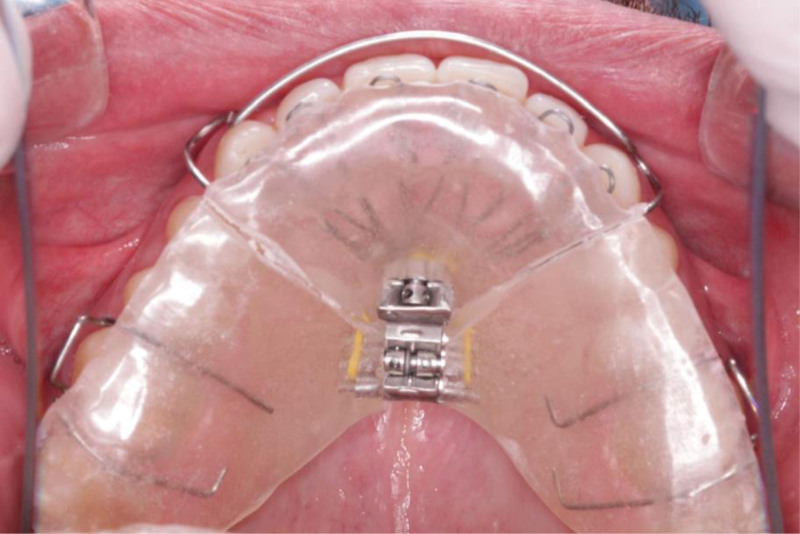
The DNA appliance.

The DNA appliance is composed of a maxillary appliance with 3 direction expansion screw that expands the palate in the anteroposterior and horizontal directions (Fig. 2). The design includes a maxillary removable device consisting of 6 (patented) anterior 3D axial springs, a beaded pharyngeal extension, a midline screw, a horizontal screw, bilateral occlusal coverage, retentive Adam’s clasps, and a labial bow.

Patients were instructed to wear the appliance 10 to 12 h/d. Patients were instructed to wear the device for the first 3 weeks with no adjustment. After that, patients were instructed to adjust the expansion screw by a quarter turn every week. Patients were instructed to clean the device with warm soap water to dry well and keep it in a safe place when they are not using it. Patients were seen every 2 months for regular checkups and maintenance and for making follow-up dental casts to compare arch measurements before, during, and after treatment.

### 2.5. CBCT protocol

The CBCT protocol follows JUST oral radiology clinic protocol for taking CBCT.^[16,17]^ KODAK 9500 cone beam 3D system (Carestream, Rochester, NY, USA) with flat panel detector was used to acquire CBCT images. The imaging area of CBCT is a cylinder with a height of 15 to 20.6 cm and a diameter of 9 to 18 cm, providing isotropic cubic voxels with sides approximating 0.2 to 0.3 mm. Exposure parameters were set: tube voltage – 90 kV, tube current – 10 mA, and exposure time – 20 seconds. All examinations were performed by 360° rotation in the occlusal position with the patients standing and closing their teeth. They were instructed to put the tip of the tongue against the palatal surface of maxillary anterior teeth. Patients were instructed to stay still and not move while taking the CBCT.

### 2.6. CBCT analysis

CBCT analysis was done by 1 qualified radiologist (ES) at the KAUH radiology unit using the dedicated CBCT Software (Kodak CS 3D imaging version 3.8.6, Carestream). A total of 17 measurements were taken for each patient pre and posttreatment (Table S1, Supplemental Digital Content, http://links.lww.com/MD/N669). These 17 measurements can be classified into 3 sets: set A: cephalometric analysis, set B: upper airway measurements, and set C: hard palate measurements and posterior nasal apertures and choncae measurements (Figs. S1–S8, Supplemental Digital Content, http://links.lww.com/MD/N668).

### 2.7. Statistical analysis

Statistical package for social sciences (SPSS software, Version 23, IBM, Chicago, IL). Paired *t*-tests to compare the sleep study measures (AHI pre and posttreatment) and the changes in the CBCT measurements before and after the BOAT treatment. *P* < .05 was considered significant. Data were presented as mean ± SD.

## 3. Results

A total of 17 adults (9 males, 8 females) were recruited following a confirmed diagnosis of OSA. The mean age (SD) was 45.76 (10.31), the mean body mass index (BMI, SD) was 33.5 (13.43), and the mean AHI (SD) was 22 (11.7). The OSA severity distribution of the sample is shown in Table 1. The treatment duration was 15.4 ± 6.3 months. Before treatment, at the diagnosis stage, the mean AHI of the sample (n = 17) was 24.0. After treatment, the mean AHI fell by 5% to 22.8% (*P* = .019), indicating enhanced upper airway functions. Table 2 displays CBCT measurements pre and posttreatment. AHI and SPO_2_ were analyzed using Spearman correlation (Table 3). Airway measurements from the CBCT were not statistically significant despite improvement in the polysomnographic parameters.

**Table 1:** Summary of Obstructive sleep apnea (OSA) severity among the study participants.

OSA severity	n	%
Mild, AHI < 15	6	35.3
Moderate, AHI 15 to 30	8	47.1
Severe AHI ≥ 30	3	17.5

AHI = apnea-hypopnea index.

**Table 2:** CBCT measurements pre and post treatment.

Parameter	Before mean (SD)	After mean (SD)	Mean difference	*t* stat	*p*-value
AHI	22.15 (11.76)	23.70 (20.03)	−1.55	−0.354	.729
PNS	42.58 (6.43)	44.22 (7.88)	−1.64	−1.155	.271
RPD S	3.45 (1.97)	3.67 (2.11)	−0.22	−0.629	.541
RPD A	17.18 (4.92)	16.26 (5.04)	0.92	0.521	.612
MRP area	94.90 (40.64)	95.65 (49.86)	−0.76	−0.047	.963
RGD S	9.08 (3.60)	9.38 (4.52)	−0.29	−0.357	.727
RGD A	17.29 (5.67)	16.83 (6.54)	0.46	0.311	.761
MRG area	189.61 (92.35)	203.65 (103.21)	−14.05	−0.809	.434
MIMW	36.78 (3.28)	36.26 (2.37)	0.52	0.838	.418
SA PNA coronal	426.79 (162.78)	461.98 (137.99)	−35.19	−1.236	.24
SA PNA axial	199.14 (82.77)	184.78 (111.41)	14.35	0.522	.611
RtCho	2.11 (1.12)	0.95 (0.64)	1.15	3.661	.003
LtCho	1.99 (0.77)	1.58 (1.03)	0.42	0.939	.366

AHI = apnea-hypopnea index, LtCho = left chonca, MIMW = maxillary intermolar width, MRG area = minimum retro-glossal area, MRP area = minimum retro-palatal area, PNS = posterior nasal spine, RGD A = retro-glossal axial, RGD S = retro-glossal sagittal, RPD A = retro-palatal distance axial, RPD S = retro-palatal distance sagittal, RtCho = right chonca, SA PNA = surface area of the posterior nasal apertures.

**Table 3:** Analysis of the correlation between AHI and SpO_2_ using Spearman correlation.

		Δ Total AHI	DIFF_RPD_A	Diff_RGD_A	Diff_SA_PNA_coronal	Diff_SA_PNA_axial
Δ Total AHI	Correlation coefficient	1.000	.588*	.066	−.187	.137
	Sig. (2-tailed)		.035	.830	.541	.655
DIFF_RPD_A	Correlation coefficient	.588*	1.000	.721**	.198	.423
	Sig. (2-tailed)	.035		.005	.517	.150
Diff_RGD_A	Correlation coefficient	.066	.721**	1.000	.374	.556*
	Sig. (2-tailed)	.830	.005	.	.208	.049
Diff_SA_PNA_coronal	Correlation coefficient	−.187	.198	.374	1.000	.484
	Sig. (2-tailed)	.541	.517	.208		.094
Diff_SA_PNA_axial	Correlation coefficient	.137	.423	.556*	.484	1.000
	Sig. (2-tailed)	.655	.150	.049	.094	

AHI = apnea-hypopnea index, RGD A = retro-glossal axial, RPD A = retro-palatal distance axial, SA PNA = surface area posterior nasal apertures.

* Correlation is significant at the 0.05 level (2-tailed).

** Correlation is significant at the 0.01 level (2-tailed).

## 4. Discussion

CBCT has become readily available in the dental practice, allowing practitioners to receive 3-dimensional data that were not otherwise available in day-to-day practice. Its availability and the width and breadth of its view enable the examination of areas commonly not examined by the dentist, such as the upper airway. The combination of the clinical signs and symptoms, in addition to the careful examination of the CBCT, has a significant diagnostic value in identifying the risk factors of OSA, in addition to the follow-up and evaluation of treatment outcomes after treatment with oral appliances like in this study where we used the CBCT in addition to the AHI to assess treatment outcomes as in this study design. Parameters that were significantly improved by the end of the treatment period included total AHI/Per hour of sleep (*P* = .01), non-rapid eye movement- AHI (NREM-AHI, *P* = .02), desaturation index (*P* = .04), average SpO_2_ (*P* = .09), and average O_2_ while in non-REM (*P* = .04).^[15]^

Airway analysis begins at the beginning of the airway, specifically in the nasal cavity. The nasal valves are evaluated for symmetry and nasal deviation or obstruction. Significant findings include pneumatized or enlarged turbinates. The next area to assess is the oropharynx. The minimum cross-sectional area is evaluated in the sagittal plane. Additional useful measurements include the cranial base. These help assess the craniofacial growth pattern, particularly in children and adolescents. In adults, these measurements evaluate the possible etiology and identify the skeletal pattern that puts the patient at risk of developing OSA later in life.

The CBCT analysis used in this paper is classified into 3 sets: set A, cephalometric analysis, and set B, upper airway measurements; set C, hard palate measurements, posterior nasal apertures, and choice measurements. Set A: cephalometric analysis is useful for identifying malocclusions and skeletal discrepancies in OSA patients. The second set of measurements, the airway measurements, are done in the sagittal and coronal sections to measure the minimum distance and the mediolateral width of the upper airway at 2 levels: retro-palatal area and retro-glossal area. The third set helps to analyze local obstruction in the upper airway by assessing the length of the soft palate and the minimum retro-palatal area. There are 3 keywords to describe upper airway analysis: methodical, reliable, and repeatable.^[14]^ Some airway measurements were found to correlate with the presence and severity of OSA.^[18]^ An upper airway area <52 mm^2^ predicts a high risk of developing OSA, 52 to 110 mm^2^ intermediate risk, and an airway area >110 mm^2^ low risk.^[18]^

Comparison of CBCT airway measurements between patients with OSA and snorers found that the presence and severity of OSA, as measured by AHI, was associated with a narrow lateral dimension of the airway, increasing age, male sex, and the Berlin questionnaire were identified as risk factors to develop OSA.^[19]^ It was found that patients with OSA had increased airway length, smaller minimum cross-sectional area, and an elliptically shaped airway. Patients showed smaller anteroposterior airway dimensions than the control groups.^[20]^ OSA patients have reduced airway dimensions associated with airway obstruction. Therefore, airway analysis and upper airway anatomy are useful in predicting patients’ risk of developing OSA.^[20]^ Additional anatomic risk factors include the presence of a deep palatal vault, which leads to a lower tongue position and a reduction in the retro-glossal area. The increased length of the soft palate is confirmed by the Mallampati score as modified by Friedman.^[16]^

Momany et al conducted a case-control study to explore the anatomic risk factors of the upper airway for OSA in Jordanian patients. The soft palate length and narrow cross-sectional area are risk factors for OSA.^[21]^ In addition, the study found that being overweight, as assessed by the BMI, is a consistent contributing factor. It was found that the presence of torus mandibularis, narrow pharyngeal walls, and Mallampati score 4 were risk factors for OSA, in addition to a high BMI, which is consistent with our clinical findings.^[22]^

Dynamic imaging of the airway with mandibular advancement devices (MADs) reveals that MADs alter airway collapsibility and decrease extraluminal pressure, which is vital for managing OSA.^[23]^ The greatest effect of the MAD appliances was found in the retro-glossal and retro-palatal areas. The tongue protrusion and lateral expansion of the airway in the oropharynx area of the upper airway were also observed. The airway changes’ pattern and magnitude differ between oral appliances.^[23]^ Tongue-retaining devices had a greater effect than MADs because they produced more anterior tongue movement than MAD, which only produced 6 to 10 mm of mandibular advancement. It is important to be able to identify responders vs Nonresponder.^[24]^ Younger patients with a convex facial profile show favorable treatment outcomes. While patients with increased lower facial height and a vertically restricted throat seemed to worsen treatment outcomes. There is a limit in which no further increase of the mandibular advancement is useful.^[24]^

The CBCT image does not allow soft tissue contrast. Soft tissue structures such as lymph nodes, tonsils, and the pharynx appear indistinguishable. This makes the soft tissue airway boundaries easy to identify. This allows for the examination of airway symmetry and contours. An MRI image is prescribed if any pathology is suspected.^[14]^ In addition, CBCT is taken when the patient is standing in an upright position and awake. This does not replicate the dynamic airway during sleep in a supine position.^[14]^ The patient’s tongue position can affect the oropharyngeal dimensions. Therefore, a neutral tongue position is encouraged.^[14]^

## Author contributions

**Conceptualization:** Lina Khasawneh, Noor Al Mortadi, Eslam Abu-Ishqair, Basheer Khassawneh, Karem H. Alzoubi.

**Data curation:** Lina Khasawneh, Noor Al Mortadi, Eslam Abu-Ishqair, Basheer Khassawneh, Karem H. Alzoubi.

**Formal analysis:** Lina Khasawneh, Noor Al Mortadi, Eslam Abu-Ishqair.

**Funding acquisition:** Lina Khasawneh, Noor Al Mortadi, Basheer Khassawneh.

**Investigation:** Lina Khasawneh, Noor Al Mortadi, Eslam Abu-Ishqair, Basheer Khassawneh, Karem H. Alzoubi.

**Methodology:** Lina Khasawneh, Noor Al Mortadi, Eslam Abu-Ishqair, Basheer Khassawneh.

**Project administration:** Lina Khasawneh, Noor Al Mortadi, Basheer Khassawneh, Karem H. Alzoubi.

**Resources:** Lina Khasawneh, Noor Al Mortadi, Karem H. Alzoubi.

**Supervision:** Lina Khasawneh, Noor Al Mortadi, Eslam Abu-Ishqair.

**Validation:** Lina Khasawneh, Noor Al Mortadi, Eslam Abu-Ishqair, Karem H. Alzoubi.

**Visualization:** Lina Khasawneh, Eslam Abu-Ishqair, Basheer Khassawneh, Karem H. Alzoubi.

**Writing – original draft:** Lina Khasawneh, Noor Al Mortadi, Eslam Abu-Ishqair, Basheer Khassawneh, Karem H. Alzoubi.

**Writing – review & editing:** Lina Khasawneh, Noor Al Mortadi, Eslam Abu-Ishqair, Basheer Khassawneh, Karem H. Alzoubi.

## Supplementary Material


