# Supplementary material for: Cone beam computed tomography changes upon oral appliance therapy for adult patients with obstructive sleep apnea: A non-randomized clinical trial
Source: Medicine (Baltimore). 2024 Oct 4;103(40):e39923. doi: 10.1097/MD.0000000000039923 (PMC11460865; doi:10.1097/MD.0000000000039923)
Supplement: Supplementary file 1 [file medi-103-e39923-s001.docx]

| **No.** | **Measurement** | **Code** | **Unit** |
| --- | --- | --- | --- |
|  | Upper Incisor Maxillary Plane | UInc-MxPl | Deg |
|  | Lower Incisor Mandibular Plane | LInc-MnPl | Deg |
|  | Maxillary - Mandibular Plane Angle | MMPA | Deg |
|  | Facial Proportion | FP | % |
|  | Gonial angle: | Po-Go-Co | Deg |
|  | Length of soft palate in mid sagittal plane: | PNS-U | Mm |
|  | Minimum retropalatal distance in the mid sagittal plane: | RPD-S | Mm |
|  | Minimum retroglossal distance in the mid sagittal plane: | RGD-S | Mm |
|  | Minimum retropalatal medio-lateral width in the axial plane at the same level as #9 above: | RPD-A | Mm |
|  | Minimum retropalatal area in the axial plane at the same level as #9 above: | MRP-Area | mm2 |
|  | Minimum retroglossal medio-lateral width in the axial plane at the same level as #10 above: | RGD-A | Mm |
|  | Minimum retroglossal area in the axial plane at the same level as #10 above: | MRG-Area | mm2 |
|  | Minimum maxillary intermolar width at the cervical margin of the mesio-palatal cusps of the first molars: | MIMW | Mm |
|  | Surface area of the posterior nasal apertures at the level of the posterior nasal spine in the Coronal plane: | SA-PNA , coronal | mm2 |
|  | Surface area of the posterior nasal apertures at the level of the posterior nasal spine in the Axial plane: | SA-PNA;axial | mm2 |
|  | Minimum inferior nasal concha space from the septum on the right side: | RtCho | Mm |
|  | Minimum inferior nasal concha space from the septum on the left side: | LftCho | Mm |
