# Supplementary material for: Cone beam computed tomography changes upon oral appliance therapy for adult patients with obstructive sleep apnea: A non-randomized clinical trial
Source: Medicine (Baltimore). 2024 Oct 4;103(40):e39923. doi: 10.1097/MD.0000000000039923 (PMC11460865; doi:10.1097/MD.0000000000039923)
Supplement: Supplementary file 2 [file medi-103-e39923-s002.docx]

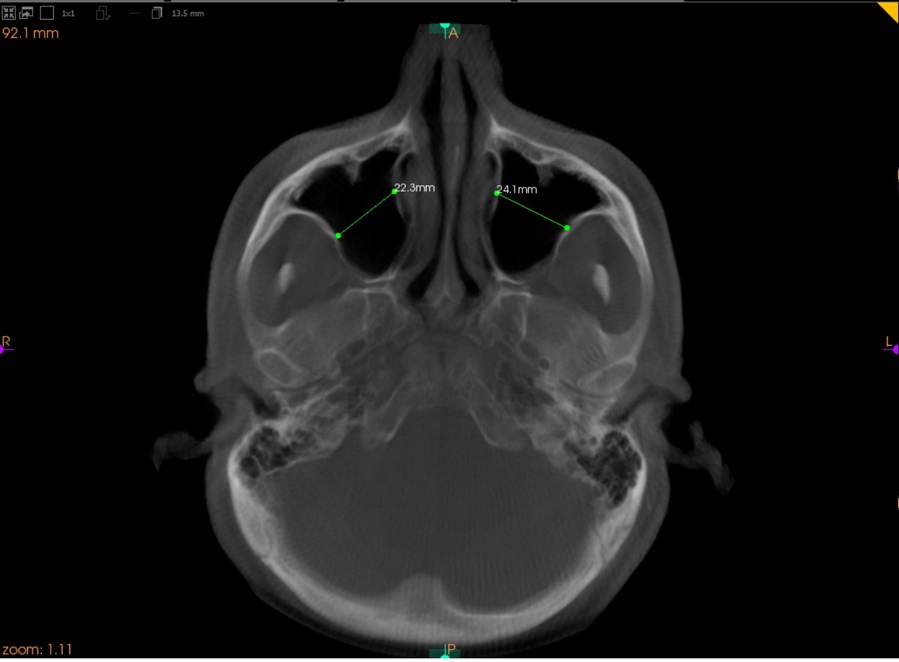


**Supp Figure 1:** Coronal assessment of nasal turbinates and maxillary sinuses.


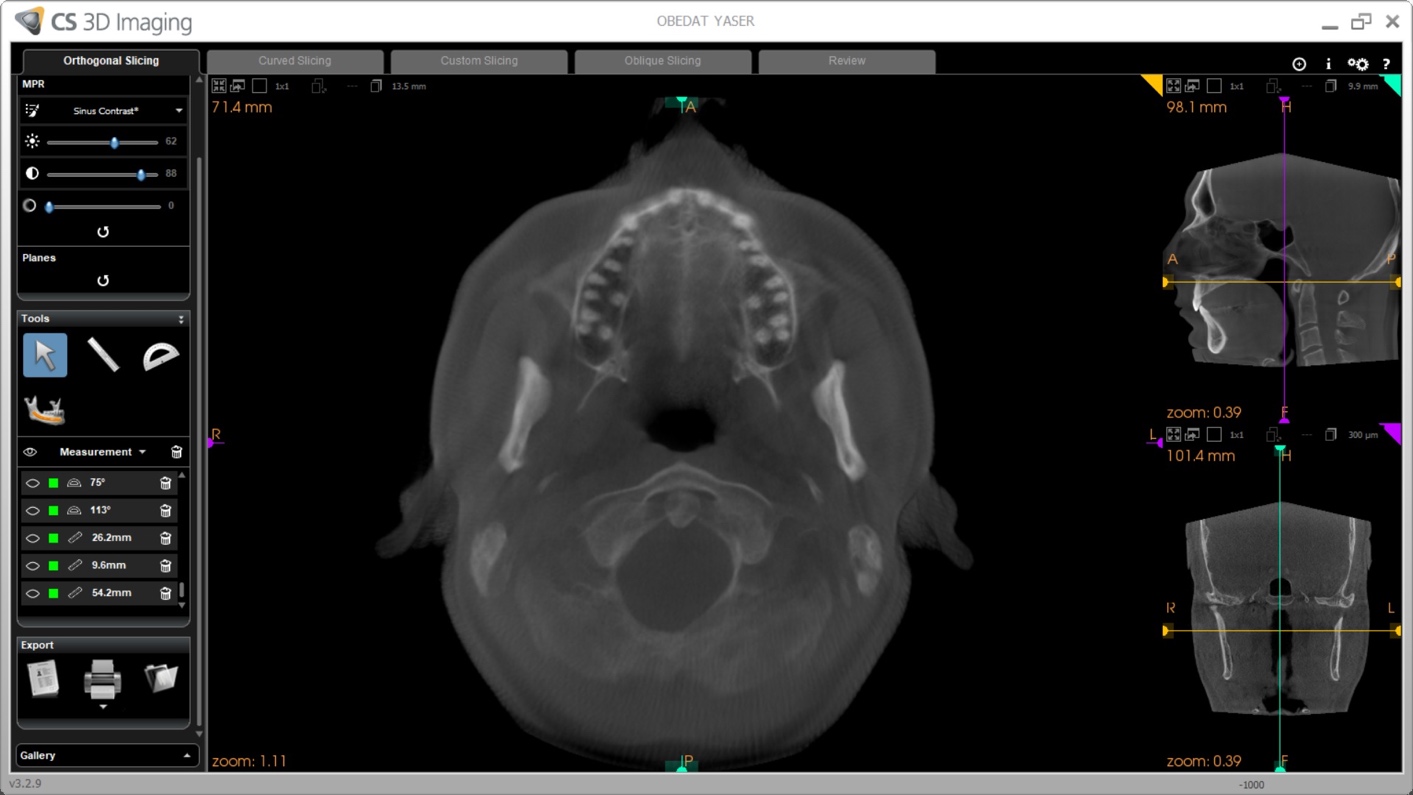


**Supp Figure 2:** Assessment of the oropharynx, the minimum cross-sectional airway measurement.


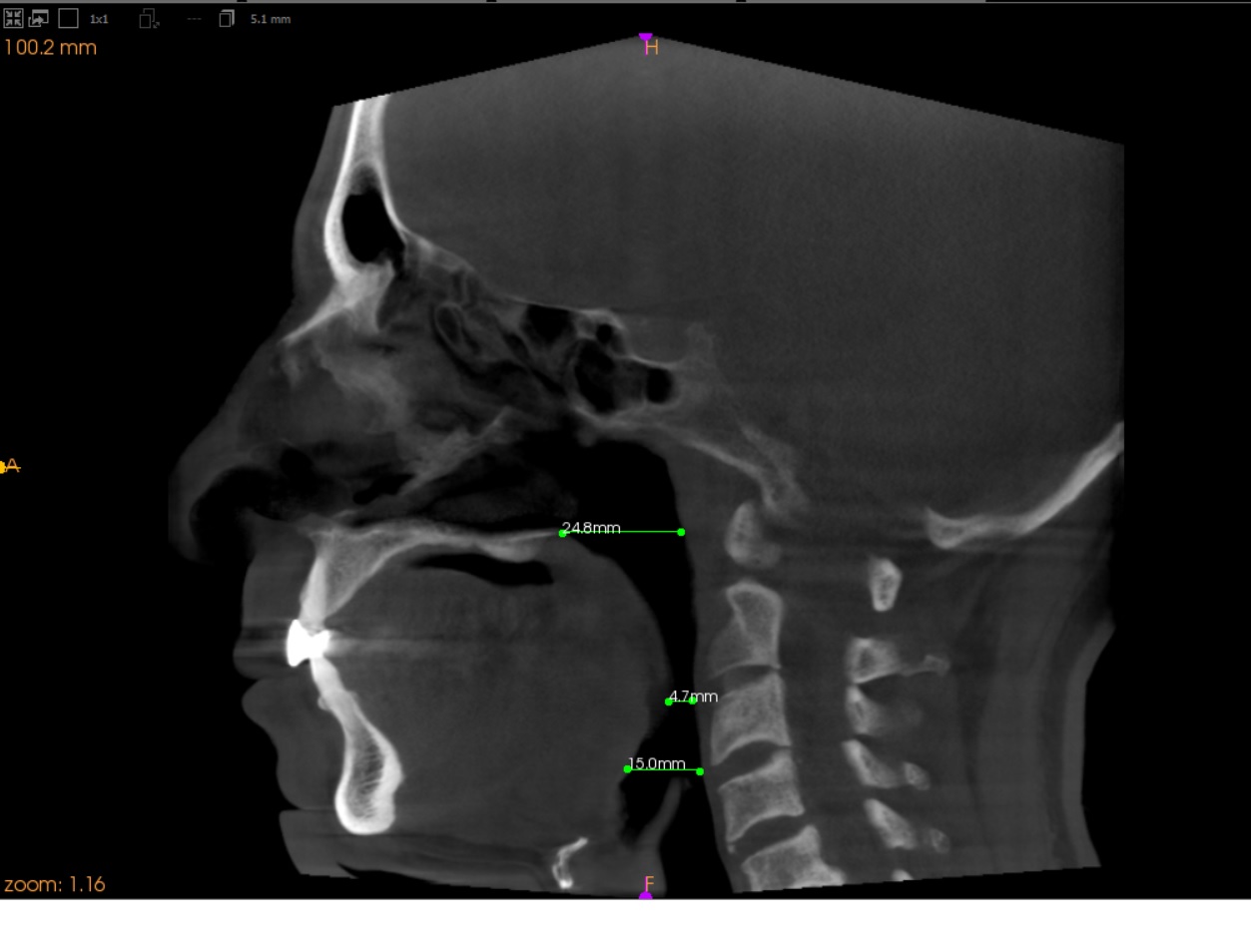


**Supp Figure 3:** The Minimum cross-sectional area of the upper airway.


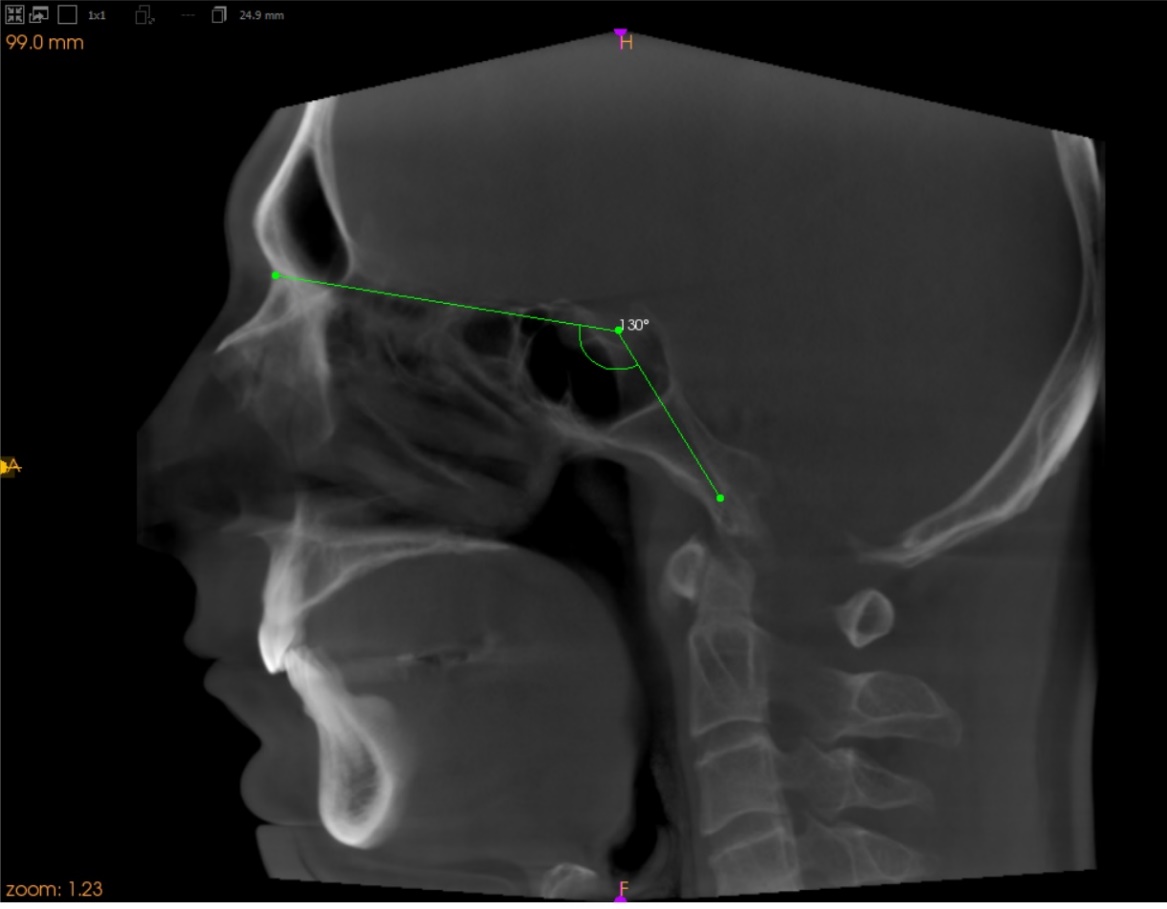


**Supp Figure 4:** The Cranial Base Angle.


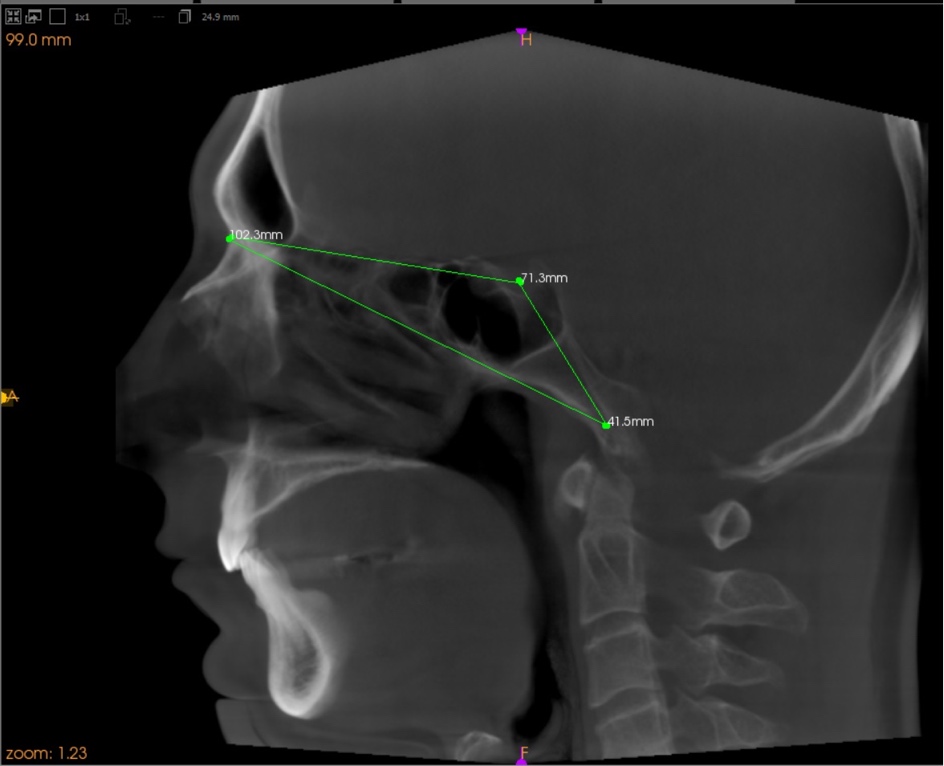


**Supp Figure 5:** The Cranial Base Length.


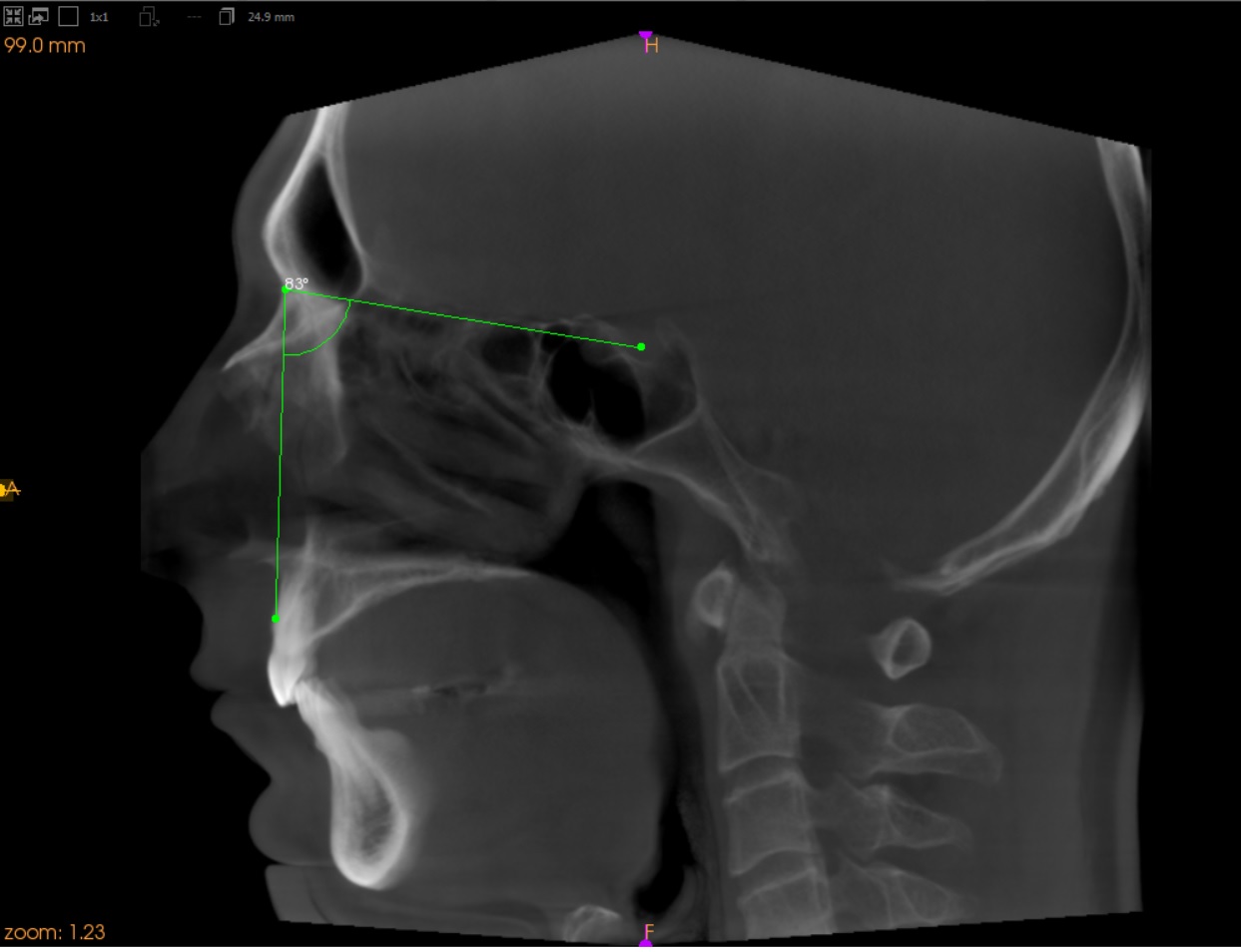


**Supp Figure 6:** The sella-nasion-point A (SNA) angle.


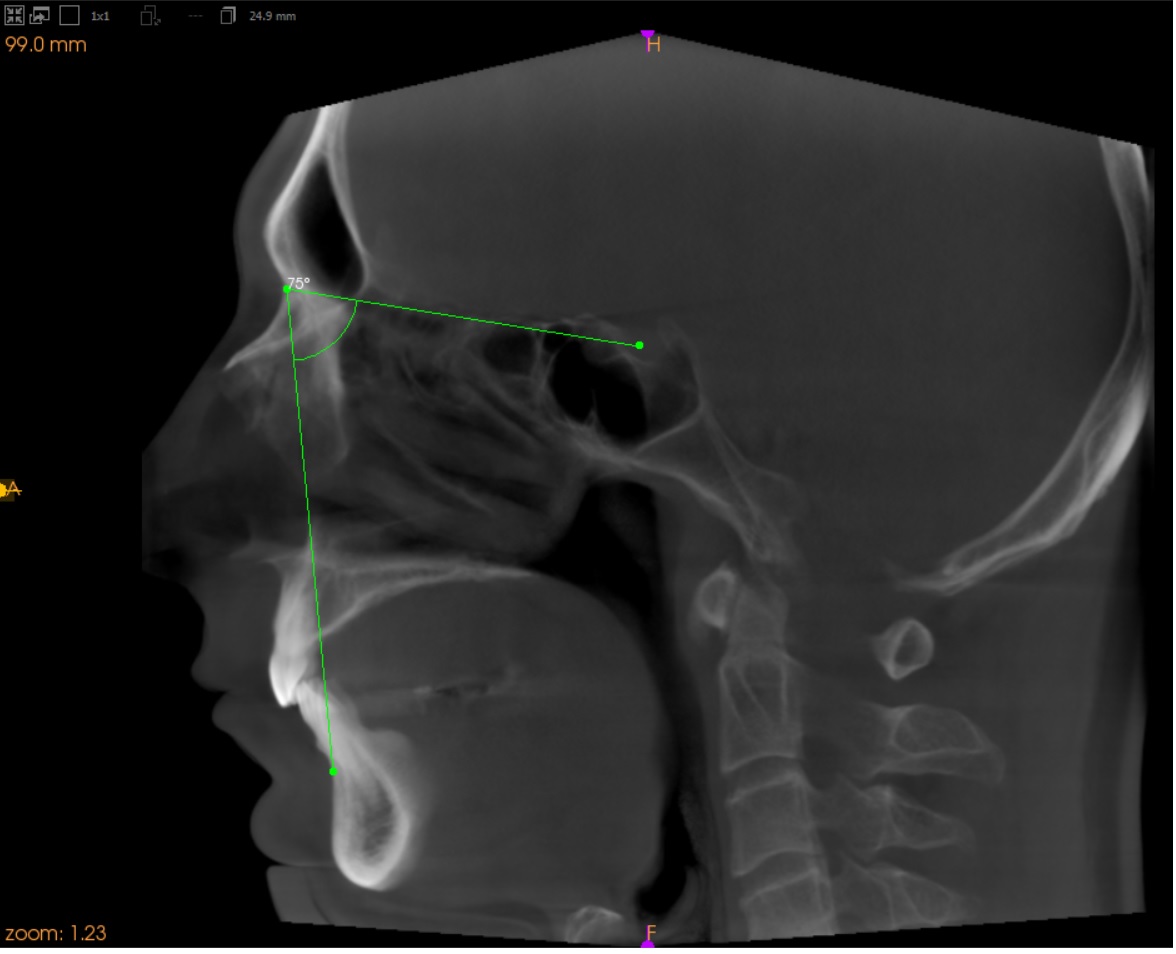


**Supp Figure 7:** The sella-nasion-point B (SNB) angle.


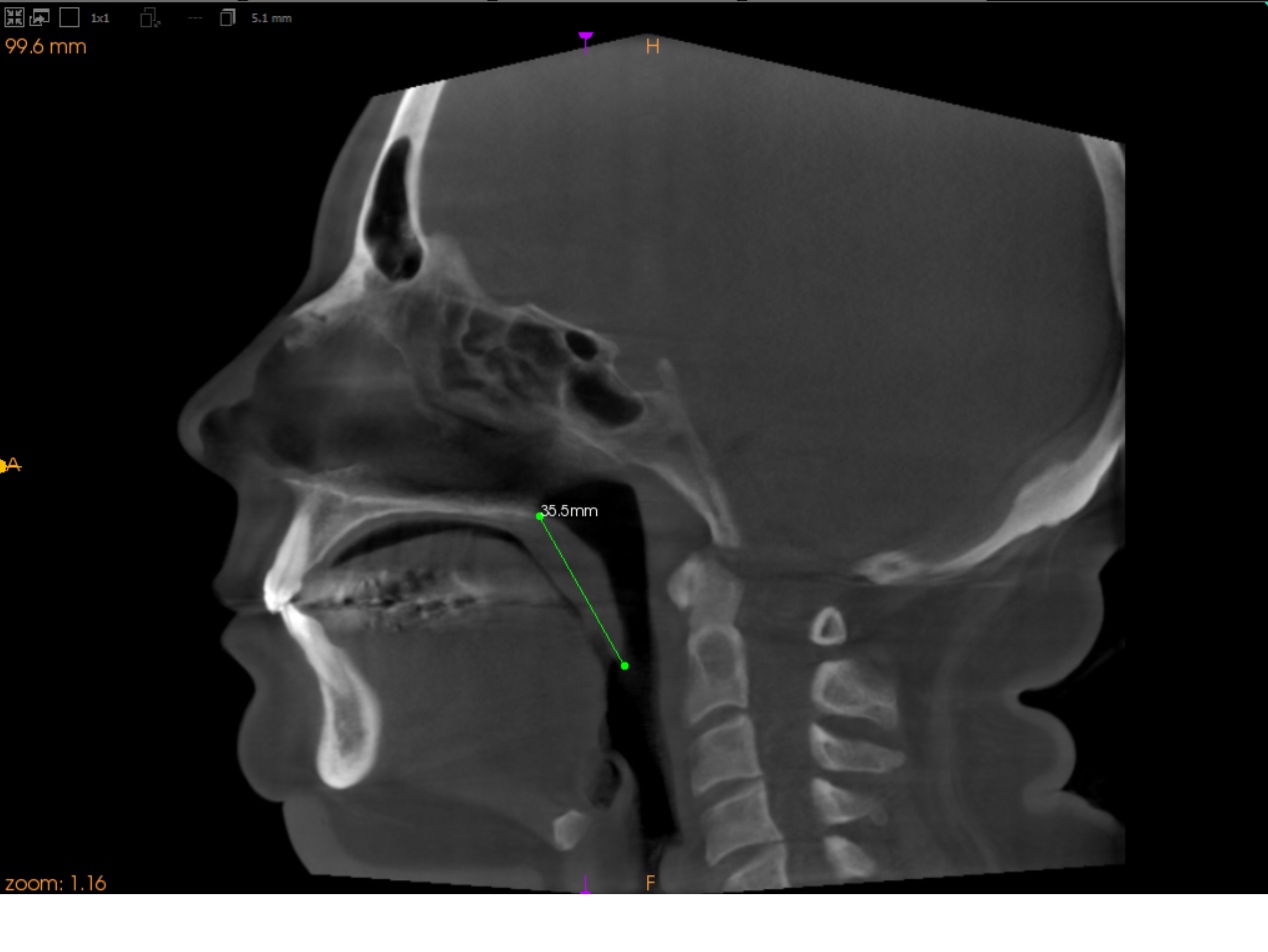


**Supp Figure 8:** The length of the soft palate.
